# The Potential Role of Passive Antibody-Based Therapies as Treatments for Monkeypox

**DOI:** 10.1128/mbio.02862-22

**Published:** 2022-10-31

**Authors:** Evan M. Bloch, David J. Sullivan, Shmuel Shoham, Aaron A. R. Tobian, Arturo Casadevall, Kelly A. Gebo

**Affiliations:** a Department of Pathology Johns Hopkins University School of Medicine; b Department of Molecular Microbiology, Johns Hopkins Bloomberg School of Public Health; c Department of Immunology, Johns Hopkins Bloomberg School of Public Health; d Department of Medicine, Division of Infectious Diseases, Johns Hopkins University School of Medicine; Albert Einstein College of Medicine

**Keywords:** passive immunization, monkeypox, plasma, antibodies, serotherapy

## Abstract

Monkeypox, a zoonosis caused by the orthopox monkeypox virus (MPXV) that is endemic to Central and West Africa, was previously linked to sporadic outbreaks and rare, travel-associated cases. An outbreak of monkeypox in 2022 has spurred a public health emergency of international concern, and this outbreak is unprecedented in terms of its scale and epidemiology. The outbreak has been focused overwhelmingly in men who have sex with men; however, the trajectory of the outbreak remains uncertain, with spread now being reported in women and children. The mortality has been low (<1%), yet the morbidity is high. Vaccines and oral antiviral agents that have been developed to protect against smallpox are available for use against monkeypox. However, the supply has been unable to match the demand during the outbreak. Passive antibody-based therapies, such as hyperimmune globulin (HIG), monoclonal antibodies, and convalescent plasma (CP), have been used against a diverse array of infectious diseases, culminating in their extensive use during the COVID-19 pandemic. Passive antibody-based therapies could play a role in the treatment of monkeypox, either as a temporizing role amid a shortfall in vaccines and antivirals or a complementary role to direct-acting antivirals. Drawing on the collective experience to date, there are regulatory, administrative, and logistical challenges to the implementation of antibody-based therapies. Their efficacy is contingent upon early administration and the presence of high-titer antibodies against the targeted pathogen. Research is needed to address questions pertaining to how to qualify HIG and CP and to determine their relative efficacy against MPXV, compared to antecedent therapies and preventative strategies.

## INTRODUCTION

Monkeypox, a zoonosis that was formerly the domain of sporadic outbreaks in Central and West Africa, is now a public health emergency of international concern (1). Previously, the largest outbreaks of monkeypox were limited to a few hundred cases; however, over 71,000 cases have been reported in the current outbreak, spanning 100 countries that were previously deemed to be nonendemic (2). The trajectory of this outbreak remains uncertain. While cases of monkeypox have been overwhelmingly focused in men who have sex with men (MSM), the end of summer heralded the reopening of schools and colleges, exposing new populations to risk. Further, the high incidence may permeate to susceptible populations, such as the immunosuppressed, in which the mortality risk is higher.

Two-and-a-half years into the historic COVID-19 pandemic, public health agencies look to treatment and prevention strategies to gain control of yet another crisis spurred by an infectious disease. Within the available armamentarium of options, passive antibody-based therapies, such as hyperimmune globulin (HIG), monoclonal antibodies (MAbs), and convalescent plasma (CP), merit consideration. Antibody-based therapies have been used for over a century to treat a myriad of infectious diseases (3–5). The scale of the COVID-19 pandemic has afforded novel insights into their procurement and clinical use (6–10). Passive antibody-based therapies could play a temporizing role amid the shortfall in vaccines and antivirals. They could also assume a complementary role to direct-acting antivirals, particularly in subsets of patients who are at risk of severe or refractory disease, such as the immunosuppressed. We review monkeypox with a specific focus on the potential roles of passive antibody-based therapies in the current outbreak.

## MONKEYPOX VIROLOGY

Monkeypox virus (MPXV) is a member of the orthopox family of *Poxviridae*, which is characterized by robust immunologic cross protection against related vaccinia and variola (smallpox) (11). During outbreaks of monkeypox attributed to clade I (formerly the Congo Basin clade) in Africa, the case fatality rate (CFR) varied from less than 5% to a high of 10% (12, 13). In contrast, the CFR associated with the less virulent clade II (formerly the West African clade), was less than 1% (13). As of October 7, 2022, only 26 deaths had been reported, yielding a CFR of 1 per 2,734 (2). The less virulent clade II has been implicated in both the 2003 outbreak in the United States of America (12) as well as the current international MPXV outbreak in 2022 (clade IIb) (14).

MPXV has about 20 diverse proteins on the surface of the mature virion (MV) and an additional 6 on the enveloped virion (EV), with a complex interaction for the wide cell and host range invasion (15). A few proteins and glycoproteins (D8, H3, A27, L1, A33, and B5 [A13]) account for neutralization activity (16). Intact vaccinia virus elicits cell and humoral responses to these and more viral molecules for protection (17). High levels of neutralizing antibodies and cellular responses can be demonstrated for 30 to 40 years after vaccination (18). The relative contribution of antibodies versus cellular responses for disease protection has not been determined (19–21).

## CLINICAL MANIFESTATIONS

The incubation period for MPXV is usually from 5 to 13 days, but it can range from 4 to 21 days (22). The most commonly reported symptoms are rash with a viral prodrome of fever, chills, and lymphadenopathy. The skin eruption generally begins a few days after the fever and may last for 2 to 3 weeks. The number of lesions varies from patient to patient, from a few lesions to diffuse skin involvement. The rash is typically concentrated on the face but often is on the palms and soles, and it can involve mucous membranes, the anus, and conjunctivae. Unlike in previously reported outbreaks, genital, rectal, or oral symptoms, including rectal pain (22%), rectal bleeding (10%), and purulent or bloody stools (21%) have been commonly reported in 2022 (23). The rash typically begins with small (2 to 5 mm) macules that evolve into papules, vesicles, and then pustules. The lesions often develop umbilication and crust over, after which the crusts dry and fall off. This typically occurs 7 to 14 days after the rash begins. The vesicular rash needs to be differentiated from other infections, including varicella, syphilis, and herpes simplex. Historically (i.e., in prior outbreaks and case reporting), monkeypox lesions developed and evolved together; however, in the 2022 outbreak, this has not necessarily been the case, and lesions have been observed to be in different stages of development (23). The rash is often painful and becomes more pruritic as the lesions crust over and begin to heal. In addition, the viral prodrome typically happens before the rash appears; again, this has not been the case in the 2022 outbreak, in which prodromal symptoms have not always preceded the rash, and, instead, the presenting symptoms could be anorectal pain, rectal discharge, or rectal bleeding. For most patients, monkeypox is a self-limited disease that resolves in 2 to 4 weeks. However, severe cases have been reported in children and in those with underlying immunodeficiencies (24). Several complications of monkeypox have been reported, including pneumonia, sepsis, and encephalitis. The most recent deaths reported from monkeypox were due to encephalitis.

## A HISTORICAL PERSPECTIVE ON MONKEYPOX

MPXV was first identified, isolated, and characterized in a cynomolgus monkey (imported from Asia to Copenhagen) from a colony of laboratory monkeys during an outbreak in 1958 (25). The first human case of monkeypox was reported in a 9-month-old boy in Basankusu Territory, Democratic Republic of the Congo (DRC) in 1970, and the isolate was sent to Moscow for confirmation (26). The index patient presented with a fever, which was followed 2 days later by a centrifugal rash, typical of smallpox. The child had not been vaccinated against smallpox. While a source was not definitively identified, bushmeat consumption was reported in the family. Humans were previously viewed as an incidental or occasional host, based on surveillance in villages in equatorial Africa, where monkeypox primarily affected young children through the trapping of animal hosts (e.g., squirrels) and bushmeat consumption (27, 28).

Subsequent to the index case, rare and sporadic cases of monkeypox have been reported in residents in West Africa and Central Africa, as well as in individuals with recent travel history to countries that were suspected to be endemic (29–32). Prior to 2022, outbreaks, albeit rare, had also been reported (Table 1), most notably in the DRC (1996 to 1997) (33), Nigeria (2017 to 2018) (34), and the United States (US) (2003) (35). The latter was ascribed to the importation of Gambian giant rats or dormice from Ghana, which in turn had contact with North American prairie dogs that were sold as pets by an Illinois animal distributor (35). An editorial note highlighted the hazards of the exotic pet trade, whereby nonindigenous zoonotic pathogens are introduced to naive animal populations (35). A study noted the increasing rates of clinical monkeypox in the DRC, citing the lack of smallpox vaccination as a substantial risk factor for clinical monkeypox (11).

**TABLE 1:** Past cases and outbreaks of monkeypox in humans

Location(s)	Year(s)	Major findings	Reference
Democratic Republic of the Congo	1970	• First reported human case of monkeypox • 9-month-old child with suspected smallpox • No contacts established • Bushmeat consumption reported by family, but direct contact not confirmed	26
Liberia, Nigeria, and Sierra Leone	1970 to 1971	• 4 cases in Liberia and 1 each in Nigeria and Sierra Leone • 3 males and 3 females • Median age of 5 years (range: 4 to 24 years)	29
Katako-Kombe and Lodja health-care zones, Kasai Oriental, Democratic Republic of the Congo	1996 to 1997	• 419 cases: 304 (73%) probable and 115 (27%) possible monkeypox cases • 85% in children <16 years of age • 5 deaths (case fatality ratio: 1.5%) • Attack rate (AR) 0.3 to 1.1 per 1,000 population	33
United States of America	2003	• 71 cases spanning six states: Wisconsin (*n* = 39), Indiana (*n* = 16), Illinois (*n* = 12), Missouri (*n* = 2), Kansas (*n* = 1), and Ohio (*n* = 1) • Median age of 28 years (range: 1 to 51 years) • 55% female • Majority had exposure to prairie dogs; all confirmed cases were associated with an animal distributor in Illinois; prairie dogs were infected through contact with Gambian giant rats and dormice from Ghana	35
Nigeria	2017 to 2018	• 122 confirmed or probable human cases spanning 17 states; 7 deaths (case fatality ratio: 6%). • Human-to-human transmission • Largest outbreak prior to 2022	34
Israel	2018	• Israeli resident returning from Nigeria • Disposed of 2 rodent carcasses • No secondary transmission	30
United Kingdom	2018	• Two cases, both with recent travel to Nigeria • Consumption of bushmeat and contact with an individual with a “monkeypox like rash”	31
Singapore	2019	• Traveler from Nigeria • Reported ingestion of bushmeat • No secondary transmission	32
Multinational	2022 to present	• Human outbreaks in multiple countries; unlike prior outbreaks, reporting focused on countries previously considered to be nonendemic • Human-to-human transmission • 98% in men who have sex with men • 75% white and 41% HIV positive	37

## MONKEYPOX IN 2022: A GLOBAL HEALTH CRISIS, LONG IN THE MAKING

In 2022, the World Health Organization (WHO) reported that monkeypox was endemic in several African countries, with the highest number of cases emerging in the DRC (36). A global outbreak was first reported in May of 2022, with the initial cases noted in England. Those cases had not been associated with recent travel to an area of endemicity or a known close contact. Similarly, cases that were devoid of a travel history were subsequently reported in young men in other parts of Europe (37). The first case in the US was reported on May 17, 2022 (38). The WHO declared this outbreak to be a public health emergency on July 23, 2022 and the US Department of Health and Human Services declared a public health emergency on August 4, 2022 (39). Between May 17 and July 22, 2,891 cases had been reported in 43 states, the District of Columbia, and Puerto Rico, with a rapid increase in July of 2022 (23). Of those cases, nearly all were in men (99%). The majority of the cases for which there were data were locally acquired (74%), and among the cases for which race and gender were reported, over half of the cases were in black persons (26%) or Hispanic persons (28%), and approximately two in five were in whites (41%). The overwhelming majority of cases were among MSM.

## RESERVOIRS AND MODE(S) OF TRANSMISSION

A natural reservoir for MPXV has yet to be identified, but numerous animals have been shown to be susceptible to infection, which is maintaining transmission in and around humans. Animals in which MPXV has been isolated include nonhuman primates, tropical squirrels, dormice, prairie dogs, shrews, hedgehogs, and anteaters (28, 40, 41). Risk factors to humans, gleaned from past studies in “endemic” countries, include sleeping in the same room or bed as someone with monkeypox and using the same plate or cup (42). Contact with infected animals through trapping, processing, or consumption of bushmeat has also been suggested (28, 41). The modes of transmission include direct contact with lesions and respiratory droplet spread (43). Droplet spread appears to be a comparatively minor route of transmission, both in past outbreaks in Africa and in the current outbreak. In the 2022 outbreak, monkeypox has not been designated a sexually transmitted infection (STI) explicitly, having been instead labeled as “sexually associated”, despite evidence that supports sexual acquisition, such as the recovery of MPXV from semen and anorectal swabs, an association with HIV and other STIs, and recommended mitigation measures that target sexual practices (44–48). Animal models have been developed using nonhuman primates (e.g., baboons) and rodents (e.g., North American prairie dogs), and these demonstrate multiple modes of transmission (49, 50).

## DIAGNOSTICS

The CDC currently classifies potential monkeypox cases as suspected, probable, or confirmed (51). Monkeypox should be suspected in those with the characteristic rash and in those who have one or more of the epidemiological criteria (Table 2) and have a high clinical suspicion of monkeypox (i.e., a rash similar to that encountered in syphilis, herpes, and/or varicella zoster). Confirmed cases are based on the presence of MPXV DNA being observed via polymerase chain reaction (PCR) or next generation sequencing or on a clinical specimen or isolation of MPXV in culture from a clinical specimen (51). A probable case has neither suspicion of orthopox exposure nor the presence of orthopox DNA (i.e., via PCR) or orthopox virus on electron microscopy or immunohistochemical staining or detectable anti-orthopox virus IgM antibody levels 4 to 56 days after rash onset.

**TABLE 2:** Epidemiologic criteria within 21 days of illness onset for monkeypox per the CDC

Epidemiologic criteria
Reports having contact with a person or people with a similar appearing rash or who received a diagnosis of confirmed or probable monkeypox OR
Had close or intimate in-person contact with individuals in a social network that was experiencing monkeypox activity, including men who have sex with men (MSM) who met partners through an online website, digital application (“app”), or social event (e.g., a bar or party) OR
Traveled outside the United States to either a country with confirmed cases of monkeypox or where monkeypox virus is endemic OR
Had contact with a dead or live wild animal or exotic pet that is an African endemic species or used a product derived from such animals (e.g., game meat, creams, lotions, powders, etc.)

The diagnosis should also be suspected in those with genital or rectal ulcers, tenesmus, or lesions consistent with a vesicular rash. To reduce the risk of transmission, it is essential to implement appropriate infection prevention as soon as the diagnosis is considered. Diagnostic testing includes swabbing lesions from 2 to 3 distinct lesions on different areas of the body and sending them to commercial, public health, or locally available laboratories for PCR testing. Although the lesions do not need to be unroofed, clinicians should confirm requirements pertaining to specimen collection and transportation with their local laboratory or public health department. Serologic testing to support the diagnosis is possible but is generally coordinated with public health laboratories. Of note, the CDC enzyme-linked immunosorbent assay (ELISA) detects IgM and IgG approximately 5 to 8 days (respectively) after rash onset (52).

## PREVENTION

### Vaccination.

Immunization against MPXV relies on vaccinia-based products that were primarily developed for the prevention of smallpox. Historically, individuals who were vaccinated against smallpox (variola) had substantial cross-protection against MPXV (53–55). In those who had previously been vaccinated against smallpox, there was an 85% reduction in the risk of monkeypox following contact with an MPXV-infected individual (56). Vaccination strategies include postexposure prophylaxis, ideally administered within 4 days of contact with an infected person, and preexposure prophylaxis for those at a high risk for exposure to the virus (57).

The two currently available vaccine products are JYNNEOS (also known as IMVANEX and IMVAMUNE) and ACAM2000. There are limited data regarding the efficacy of either vaccine in the current outbreak. JYNNEOS contains a modified, nonreplicating virus (modified vaccinia Ankara) and is approved in the US for the prevention of MPXV (58). It is administered as two subcutaneous injections that take place 4 weeks apart. Its efficacy was inferred from immunologic studies that demonstrated an immune response similar to that of live vaccinia products, such as ACAM2000 (57). As a replication-incompetent vaccinia virus, it is safer in immunocompromised individuals and in those with atopic dermatitis. As with many other vaccines, protective vaccine responses are expected to be reduced among immunocompromised individuals. Recently, the FDA issued an alternative dosing regimen in response to the public health emergency (59), based on data from 2015 that suggests that intradermal dosing was noninferior to standard subcutaneous dosing (60).

ACAM2000 is a replication-competent, live virus vaccine that is approved for the prevention of smallpox (61). It is not approved for MPXV but may be used under an expanded access investigational new drug (EA-IND) protocol. Its use is reserved for cases in which the replication-defective JYNNEOS is contraindicated. As with previous generation vaccinia products (e.g., Dryvax), ACAM2000 is administered via multiple skin pricks using a bifurcated needle, with the goal being to elicit a localized cutaneous infection (“take”). If feasible, ACAM2000 should be avoided in individuals with immunosuppression, pregnancy, atopic dermatitis, and exfoliative skin conditions, as well as in those whose close contacts include such individuals.

### Other.

In addition to vaccines, engaging in safer sexual practices can help reduce the transmission of MPXV. The CDC has recommended against having sex with anyone who has monkeypox symptoms and taking a temporary break from activities that increase exposure to MPXV until 2 weeks after the second vaccine dose (62). A recent online survey of gay, bisexual, and other men who have sex with men in August of 2022 demonstrated that many MSM are changing their sexual practices to reduce their risk of monkeypox, including reducing their number of sexual partners, reducing one-time sexual encounters, and reducing sex with partners met via dating apps or at sex venues (63).

## TREATMENT

### Antivirals.

Current treatment options for monkeypox include the small molecule antiviral drugs tecovirimat (TPOXX or ST-246), cidofovir (Visitide), and brincidofovir (Tembexa). None of these are specifically approved for monkeypox, having been developed instead for the treatment of smallpox. The Placebo-Controlled Randomized Trial of Tecovirimat in Non-Hospitalized Monkeypox Patients (PLATINUM) and the Study of Tecovirimat for Human Monkeypox Virus (STOMP) are seeking to evaluate the safety and efficacy of Tecovirimat in monkeypox, specifically (64, 65). Efficacy is best when treatment is started as soon as is feasible after exposure. For example, in animal studies, treatment with tecovirimat within 4 days of exposure reduced the severity of illness. Survival was 100% in animals when used within 5 days of exposure but declined to 50% when started at 8 days after exposure (66). Both *in vitro* and animal studies suggest that a combination therapy (e.g., tecovirmat and brincidofovir) is superior to monotherapy (67). The small molecule antiviral drugs have the potential for the development of resistance (68). This may be particularly relevant to immunocompromised individuals with poxvirus infections and provides a rationale for combination therapy in such patients (69).

Tecovirimat is an inhibitor of the orthopoxvirus VP37 envelope wrapping protein and is approved for the treatment of smallpox (66, 70, 71). It is available in oral and intravenous forms. Tecovirimat can be used for monkeypox through an expanded access investigational new drug (EA-IND) protocol. Animal studies have demonstrated this drug’s efficacy in MPXV infections (72). Clinical evidence for use in people is limited to small observational studies. In one report, treatment appears to have shortened the duration of symptoms and viral shedding, compared to the results of patients who did not receive the drug (73). The main side effects of tecovirmat include headaches, gastrointestinal symptoms, and pain at the infusion site if administered intravenously.

Cidofovir (Vistide) is an antiviral that inhibits DNA replication and possesses a broad range of activity. It is approved for the treatment of cytomegalovirus (CMV) retinitis. Its efficacy against MPXV has been demonstrated in animal models (74). Cidofovir has been used both intravenously as well as topically to treat monkeypox, but its impact on infection is unknown (37). Intravenous cidofovir is associated with substantial renal and ocular toxicities (uveitis and iritis), but the risk for nephrotoxicity can be reduced by coadministration with probenecid.

Brincidofovir (Tembexa) is a modified version of cidofovir with good oral bioavailability and reduced nephrotoxicity. It is approved for the oral treatment of smallpox. Its efficacy has been demonstrated in animal models of MPXV infection (75). There is limited literature on its clinical use in monkeypox. In one series, three patients received oral brincidofovir at a dose of 200 mg once weekly. All three developed elevated liver enzymes, resulting in the cessation of therapy, and there was no clear evidence of efficacy (73). At this time, brincidofovir is not available from the US strategic national stockpile.

### Passive antibody-based therapies.

Antibody therapies are known to be effective against poxviruses. Vaccinia immune globulin (VIG) is an approved medication for complications following vaccinia virus immunization, illustrating the efficacy of antibodies against this class of viruses. Poxviruses, such as variola virus and MPXV, are antigenically similar, such that antibodies elicited against a vaccinia virus confer protection against monkeypox (76). Thus, VIG could theoretically have a role in monkeypox therapy; however, clinical data pertaining to its use against MPXV are not available (77). Evidence for the efficacy of antibody therapy against monkeypox comes from animal models, in which the passive administration of monoclonal antibodies has been shown to be protective against monkeypox in a marmoset animal model (78). As the monkeypox outbreak spreads, most individuals are expected to recover, resulting in a large pool of convalescent individuals who have antibodies that may be used therapeutically. Recent experience with the use of COVID-19 CP affords valuable insights in this regard.

**(i) Vaccinia gamma-globulin/hyperimmunoglobulin.** Smallpox and VIG or gamma-globulin preparations have been successfully utilized for smallpox prophylaxis and in immunodeficient settings to reverse severe/lethal manifestations of inadvertent live virus vaccination, such as eczema vaccinatum, gangrenous vaccina, fetal vaccinia, or postvaccination encephalitis for almost 100 years (79, 80). Single monoclonal antibodies are protective in select animal models but have not been clinically validated in humans (78, 81–83).

The only reported randomized clinical trials of VIG sourced after vaccinia vaccination were undertaken for smallpox prophylaxis. In the largest study by Kempe et al., there were 5/326 (1.5%) cases of smallpox in the VIG treated arm and 21/379 (5.5%) cases in the placebo arm, yielding relative and absolute risk reduction values of 73% and 4%, respectively (84). Kempe noted that 5 mL of the final hyperimmune VIG approximated the antibodies present in about 250 mL of plasma (85). Children received 5 mL of the gamma-globulin, and adults received 10 mL. A summary of all 4 of the VIG smallpox prophylaxis studies was reported by Wittek, with 9/451 new cases in the vaccination plus VIG group versus 50/558 in those who underwent vaccination alone (79).

While there are no randomized clinical trials for the treatment of severe vaccinia complications, there is a century of cumulative experience pertaining to the use of serum or vaccinia gamma-globulin preparations to reverse most clinical cases (86–88). In the 2022 outbreak of monkeypox, serious complications are to be expected in immunosuppressed individuals, which may mimic the earlier descriptions of the vaccinia postvaccination complications in the immunosuppressed.

**(ii) Monoclonal antibodies.** Monoclonal antibodies (MAbs) have been proposed to play a greater role in prophylaxis and/or the treatment of infectious diseases (89). Their extensive use during the COVID-19 pandemic has highlighted both their strengths and their limitations, particularly in the context of an emerging infectious disease. MAbs are highly targeted (e.g., target a specific epitope) and can be highly effective in reducing viral loads, the severity of symptoms, and the risk of hospitalization (90–92). However, being highly targeted is also a major limitation in that viral evolution may be quick to render MAbs ineffective. This is particularly the case with pathogens that are prone to high rates of mutation, as has been the case with SARS-CoV-2. Even with the combined use of several MAbs (93), the durability of the effect was short, given the diminished effects against successive variants and subvariants of the ancestral virus (94, 95). Despite advances in manufacturing, the timeline to availability and the implementation of MAbs for SARS-CoV2 was slow, relative to the acuity of need imposed by the pandemic. For example, bamlanivimab, the first approved MAb for clinical use against SARS-CoV2, took almost a year before becoming available (96). Under different circumstances, that might have been viewed as being highly favorable. High cost is another disadvantage. Coupled with their short durability of effect, MAbs are not financially attractive. They also need to be administered parenterally, although this is a shared limitation with the other passive antibody-based therapies. Germane to MPXV, although its mutation rates are unlikely to match that of SARS-CoV-2, MAbs are unlikely to play a major role in treatment, given the aforementioned reasons, allied with the low mortality of monkeypox.

Nonetheless, the cost of MAb treatments has improved over the course of the COVID-19 pandemic. Further, given the duration of effect of MAbs, they may be used to complement antivirals and convalescent plasma therapy. MAbs can also be modified to improve their durability. In short, while MAbs are unlikely to be introduced in the short-term to treat monkeypox, they could have a greater role in future outbreaks. The lessons learned from COVID-19, pertaining to the evaluation and deployment of MAbs emphasizing the need for correlates of immunity and appropriate randomized trials, remain relevant, independent of the virus being considered.

**(iii) Convalescent plasma.** CP for orthopox infections has not been described in published literature because of the availability of vaccinia gamma-globulin or VIG preparations. Likewise, CP has not been evaluated for MPXV, given the availability of alternative treatments, such as direct-acting antivirals, vaccination, and VIG. Nonetheless, CP for monkeypox could be studied in a target population in need: those individuals who are at a high risk of disseminated disease by virtue of their impaired immunity. Given the recency of the 2022 monkeypox outbreak, we lack information on the pathogenesis and prognosis of the disease in most types of immunosuppressed conditions. Nonetheless, we know that some patient populations, such as those with immune suppression due to HIV infection, may be at risk of severe, disseminated disease. Given the experience with COVID-19, the efficacy of monkeypox CP could be tested in high-risk populations to determine its effects on disease progression and viral load.

The efficacy of CP is contingent upon it being used early and with sufficient immunoglobulin that is specific to the targeted pathogen (97). If CP were to be employed to treat monkeypox, its evaluation must avoid the pitfalls of many of the studies of CP use against COVID-19. Specifically, numerous randomized controlled trials (RCTs) employed CP with an insufficient antibody content or too late in the disease process (98). Therefore, studies of CP in monkeypox should be designed to evaluate the administration of CP as close to the onset of symptoms as possible, immediately after randomization, and should restrict use to only the highest titer plasma. Unlike COVID-19, in which a reduction in mortality was the most relevant outcome, this is neither realistic nor relevant in monkeypox, since nearly all individuals recover.

## DELINEATING THE ROLE OF PASSIVE ANTIBODY-BASED THERAPIES FOR MONKEYPOX

There are important differences between monkeypox and COVID-19 that impact the roles of passive antibody-based therapies. Foremost, unlike COVID-19 at the start of the pandemic, there are antecedent vaccines and antiviral agents for monkeypox. This alone shifts the emphasis to prioritizing access to those measures, whereby passive antibody-based therapies could be complementary or could play a role in settings in which alternative strategies are unavailable. Second, although the morbidity from monkeypox is high, the mortality is low. This argues for a selective approach, rather than expanded population-based access, as was the case with COVID-19. Third, the lessons of COVID-19 pertaining to passive antibody-based therapies have yet to be tested against a different agent. While plausible or even probable, the research has yet to be undertaken. Experience from COVID-19 suggests that the early administration of high-titer CP is necessary for optimal effect (6–8, 10, 99). In contrast, CP was not shown to be effective either as a postexposure prophylaxis or as a treatment in late-stage disease (9, 100, 101).

## FUTURE DIRECTIONS

There are many foundational questions that still need to be addressed. These include the determination of the relative effectiveness of tecovirimat and other oral therapies versus VIG versus MPXV CP versus MPXV hyperimmune globulin against severe monkeypox, particularly in vulnerable populations, such as the immunosuppressed. Of note, nearly half of all of the cases of monkeypox in the 2022 outbreak have been among men with HIV coinfection (23). Given the low rate of mutation of human monkeypox virus compared to SARS-CoV-2, the use of a monoclonal antibody product that is broadly effective against orthopox viruses (panorthopox) may be useful in future outbreaks. There are also logistical questions pertaining to the production and distribution of the different products from VIG to MPXV CP.

## CHALLENGES PERTAINING TO PASSIVE ANTIBODY-BASED THERAPIES

There are a number of factors that could impede the adoption of antibody-based therapies for monkeypox and other emerging infectious diseases. For one, there are formidable regulatory and administrative barriers to the approval, collection, and distribution of a “new” blood product or derivative. Their approval is contingent upon the establishment and meeting of a qualification standard. Standards for plasma-based therapies that are specific to MPXV have yet to be developed. This is not trivial and requires foundational studies to determine what constitutes a high-titer plasma, what tests can be used to verify a titer, and how clinical assay results correlate with formal viral neutralization testing. A comparative analysis between automated assay and neutralization (or a surrogate, such as a viral reporter assay) is critical to avoid the distribution of low-titer CP that could later be shown to be ineffective (i.e., as occurred in the early compassionate and investigational uses of CP for COVID-19).

Second, drawing on the experience of COVID-19, there remains a difference of opinion among the major stakeholders (e.g., the World Health Organization, the US Food and Drug Administration [FDA], the National Institutes of Health [NIH], the Infectious Disease Society of America [IDSA], the Association for the Advancement of Blood and Biotherapies [AABB]) as to the role of CP, one of the major antibody-based therapies (102). Third, while much is known about the scaling of CP and hyperimmune globulin (HIG) (6, 103), it is uncertain as to how this might apply to monkeypox. More practically, the unprecedented blood shortages and adaptation to the challenges of the past two-and-a-half years has left blood collection centers understaffed and underfunded such that investment in derivatives will need incentivization amid the pandemic fatigue. Even if collection could be initiated, an outpatient infrastructure would need to be developed or adapted to another highly infectious agent (104). There are additional considerations surrounding the minimization of stigma, given the overwhelming focus of the monkeypox pandemic in the MSM community. Fourth, time is an issue. CP can be scaled rapidly, but HIG still relies on the pooling of large numbers of donations, and manufacturing transpires over months, rather than days or weeks. MPXV does not have the same rate of mutation as SARS-CoV-2, meaning that immunoglobulin that is produced now or in the near future could still prove beneficial in future outbreaks.

Finally, there is a longstanding (and notably controversial) deferral of MSM from blood donation (105). The length of deferral varies by country; in the US, the policy currently stands as a 3-month deferral since the last sexual act, meaning that almost all of the convalescent individuals would be ineligible to donate, based on the epidemiology of the outbreak (106). Drawing on the experience of COVID-19, CP is optimally collected early in the convalescent period, when MSM donors are still likely to be ineligible, particularly given that sexual transmission is a major mode of acquisition of MPXV. A potential precedent could offer a solution. While never formally implemented, a variance was approved by the US FDA Blood Product Advisory Committee in 2019 to allow for the deferral period to be waived for MSM platelet donors, contingent upon those products undergoing pathogen reduction (PR) via an approved technology (107). Of note, photochemical inactivation has demonstrated an over 4.7 log reduction in vaccinia in platelet spiking studies, suggesting efficacy against other pox viruses (108). In theory, a similar variance could be applied to collection of plasma, as long as antibody function remains intact. While plausible, the regulatory and operational complexity is high. That is, this approach is unlikely to be entertained for monkeypox.

### Conclusion.

The 2022 monkeypox pandemic is yet another example to underscore the need for a proactive response to infectious diseases. Passive antibody-based therapies offer an additional tool in the infectious disease armamentarium, given their ability to be mobilized rapidly, pending the availability of other preventative (e.g., vaccines) and/or treatment (e.g., antiviral agents) strategies. As illustrated by monkeypox, even when an antecedent vaccine is available in the setting of reemergence (rather than a case of a novel pathogen, such as SARS-CoV-2), time is needed to scale production and mobilization to meet the needs of the population. Echoing the experience with SARS-CoV-2, the limited supply of monkeypox vaccine has forced the tiering of administration to those deemed to be at the highest risk. Similarly, antiviral agents are not quick to bring to market. Further, vaccines and antivirals are notoriously slow to reach low- and middle-income countries (LMICs); in contrast, both high-income and LMICs already have the infrastructure to implement passive antibody therapy (109). While certainly not unique to monkeypox, the time to intervene in emerging infectious diseases is as early as possible. Had the foundational research on MPXV in sub-Saharan Africa been undertaken in the decades following its first recognition as a human pathogen, that is, through the establishment of a database and the study of correlates of protection, antivirals, VIG, vaccination, and vaccine effectiveness, the course of the current outbreak might have been different. This underscores the overarching need for a shift to a proactive, rather than a reflexive, approach. There is a wealth of experience from COVID-19 that can inform the collection and use of these therapies. The harmonization of efforts, particularly in the research domain, is imperative, in integrating passive antibody-based therapies into the response against emerging infectious diseases.
